# Micellized Protein Transduction Domain-Bone Morphogenetic Protein-7 Efficiently Blocks Renal Fibrosis Via Inhibition of Transforming Growth Factor-Beta–Mediated Epithelial–Mesenchymal Transition

**DOI:** 10.3389/fphar.2020.591275

**Published:** 2020-11-19

**Authors:** Seonghun Kim, Cheol-Hee Jeong, Sang Hyun Song, Jo Eun Um, Hyun Sil Kim, Jun Seop Yun, Dawool Han, Eunae Sandra Cho, Bo Young Nam, Jong In Yook, Minhee Ku, Jaemoon Yang, Man-Deuk Kim, Nam Hee Kim, Tae-Hyun Yoo

**Affiliations:** ^1^ Oral Cancer Research Institute, Yonsei University College of Dentistry, Seoul, Korea; ^2^ MET Life Science, Seoul, Korea; ^3^ Department of Oral Pathology, Yonsei University College of Dentistry, Seoul, Korea; ^4^ Department of Internal Medicine, Yonsei University College of Medicine, Seoul, Korea; ^5^ Department of Radiology, Yonsei University College of Medicine, Seoul, Korea; ^6^ Convergence Research Center for Systems Molecular Radiological Science, Yonsei University, Seoul, Korea

**Keywords:** micellized protein transduction domain-bone morphogenetic protein-7, renal fibrosis, transforming growth factor-beta, epithelial–mesenchymal transition, intervention

## Abstract

Tubulointerstitial renal fibrosis is a chronic disease process affecting chronic kidney disease (CKD). While the etiological role of transforming growth factor-beta (TGF-β) is well known for epithelial–mesenchymal transition (EMT) in chronic kidney disease, effective therapeutics for renal fibrosis are largely limited. As a member of the TGF-β superfamily, bone morphogenetic protein-7 (BMP-7) plays an important role as an endogenous antagonist of TGF-β, inhibiting fibrotic progression in many organs. However, soluble rhBMP-7 is hardly available for therapeutics due to its limited pharmacodynamic profile and rapid clearance in clinical settings. In this study, we have developed a novel therapeutic approach with protein transduction domain (PTD) fused BMP-7 in micelle (mPTD-BMP-7) for long-range signaling *in vivo*. Contrary to rhBMP-7 targeting its cognate receptors, the nano-sized mPTD-BMP-7 is transduced into cells through an endosomal pathway and secreted to the exosome having active BMP-7. Further, transduced mPTD-BMP-7 successfully activates SMAD1/5/8 and inhibits the TGF-β–mediated epithelial–mesenchymal transition process *in vitro* and in an *in vivo* unilateral ureter obstruction model. To determine the clinical relevance of our strategy, we also developed an intra-arterial administration of mPTD-BMP-7 through renal artery in pigs. Interestingly, mPTD-BMP-7 through renal artery intervention effectively delivered into Bowman’s space and inhibits unilateral ureter obstruction–induced renal fibrosis in pigs. Our results provide a novel therapeutic targeting TGF-β–mediated renal fibrosis and other organs as well as a clinically available approach for kidney.

## Introduction

Chronic kidney disease (CKD) is a serious health problem resulting from congenital abnormalities and chronic renal injury, and for which effective therapeutics are currently lacking (Bikbov et al., 2018). Renal fibrosis is the hallmark of progressive kidney injury, characterized by a tubule-interstitial fibrosis and glomerulosclerosis. Regardless of primary causes, CKD manifested as progressive renal fibrosis results in epithelial–mesenchymal transition (EMT) of tubules and glomeruli and subsequent extracellular matrix deposition. Activation of transforming growth factor-beta (TGF-β) is a well-known EMT inducer of renal fibrosis (Meng et al., 2015).

While bone morphogenetic protein-7 (BMP-7) has been studied for bone regeneration, it is now drawing great attention as a therapeutic for fibrotic diseases, including renal fibrosis. BMP-7 not only counteracts TGF-β signaling–mediated EMT via activation of SMAD1/5/8 but is also critically involved in kidney morphogenesis and development (Godin et al., 1998; Manson et al., 2015). Interestingly, mice embryos lacking BMP-7 exhibit defective kidney and eye development, indicating the importance of BMP-7 in the physiological function of kidney (Dudley et al., 1995; Jena et al., 1997). Although the effectiveness of BMP-7 for fibrosis was recognized 2 decades ago, BMP-7 therapeutics are largely lacking due to pharmacodynamic limitations, particularly in clinical settings. Animal studies have revealed that current soluble rhBMPs exhibit a half-life of 7–16 min in nonhuman primate due to rapid clearance and enzymatic degradation (Lo et al., 2012), necessitating an excess dose with initial burst release of rhBMP. Efficient drug delivery into kidney poses another obstacle in the clinical application of current rhBMP-7.

Recently, we have developed a pro-drug BMP-7 having novel mode of action fused with protein transduction domain (PTD) (Kim et al., 2014). While rhBMPs directly bind to cognate receptors, the prodrug is designed for endosomal transduction into cells (Vendeville et al., 2004; Wadia et al., 2004), and subsequent intracellular activation into active BMPs. Nevertheless, PTD-mediated cellular delivery faces several hurdles due to the limited size of hydrophobic cargos (less than 10 kd) and endosomal rescues (Kabouridis, 2003). In this study, we have developed micellized PTD-BMP-7 (mPTD-BMP-7) to enhance therapeutic application of PTD for CKD. mPTD-BMP-7 is successfully transduced into the endosomes and counteracts the TGF-β–mediated EMT process via activation of SMAD1/5/8. For clinical relevance, we have also developed interventional administration through renal artery in a pig unilateral ureter obstruction (UUO) model.

## Results

### Developmental Strategy and Preparation of Micellized Protein Transduction Domain-Bone Morphogenetic Protein-7

**FIGURE 1:**
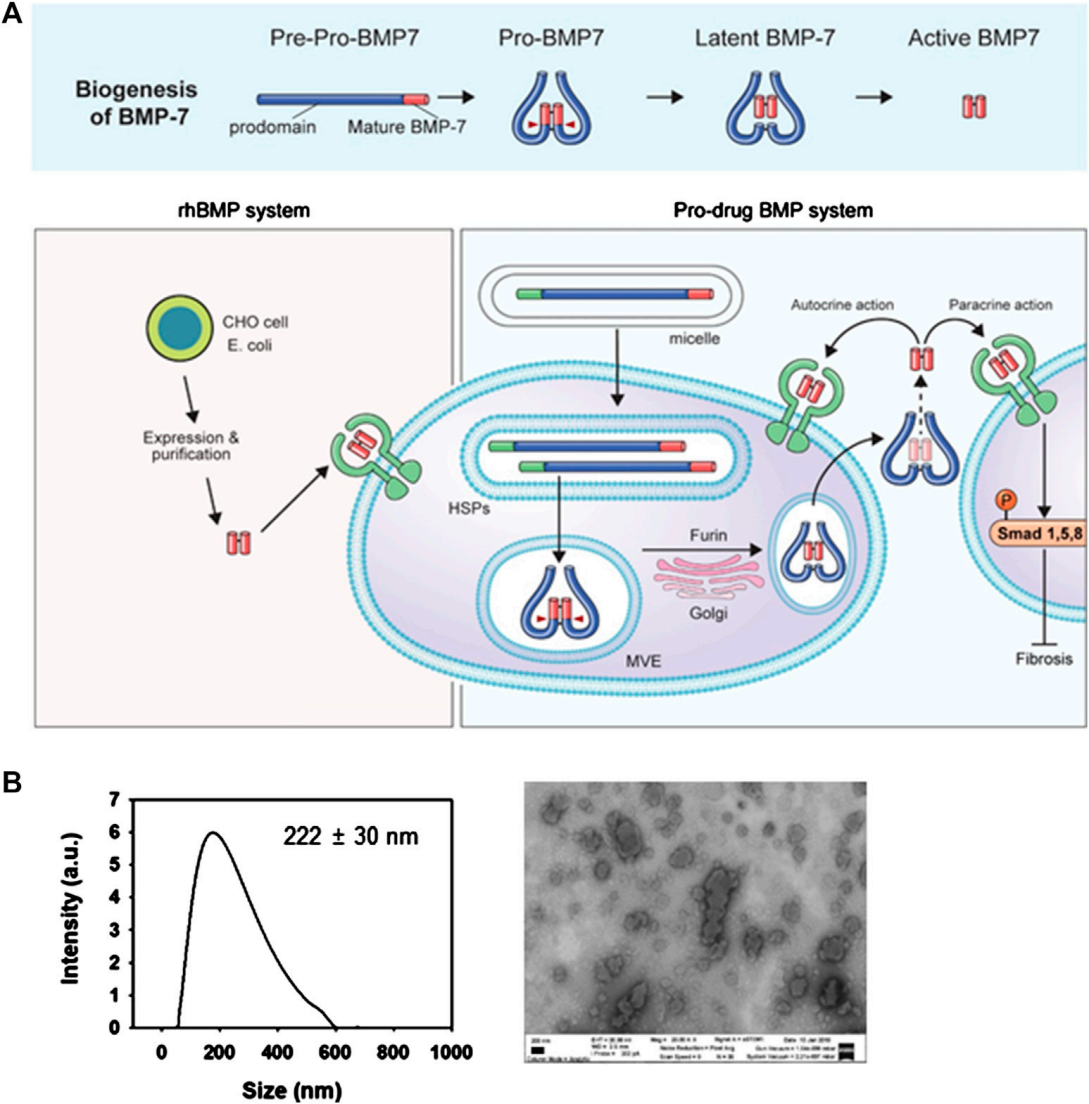
Schematic diagram and preparation of micellized PTD-BMP-7. **(A)** Schematic diagram of biogenesis of latent bone morphogenetic protein-7 (BMP-7) **(upper)**, and developmental strategy of micellized PTD-BMP-7 compared to soluble rhBMP-7 **(lower)**. **(B)** Particle size distribution detected by direct light scattering **(left)** and scanning electron microscopy **(right)**. Scale bar, 200 nm.

During the biogenesis of endogenous BMP-7, the prodomain of BMP-7 is cleaved by the subtilisin-like proprotein convertase family, a latent form of BMP-7 being secreted (Constam and Robertson, 1999). In this process, latent BMP-7 complex interacts with the N-terminal of fibrillin-1 (FBN1) microfibrils in the extracellular matrix (Gregory et al., 2005). While the rhBMPs only consist of dimeric active BMP, we have previously reported that denatured prodrug BMPs including prodomain were functional *in vivo* (Figure 1A). To increase the transduction efficiency of large cargo (i.e., 55 kd in PTD-BMP-7), we prepared mPTD-BMP-7. The typical particle size of the PTD-BMP-7 in micelle was ∼200 nm as measured by direct light scattering and scanning electron microscope (Figure 1B).

### Endosomal Transduction of Micellized Protein Transduction Domain-Bone Morphogenetic Protein-7 and Exosomal Secretion of Active Bone Morphogenetic Protein-7

**FIGURE 2:**
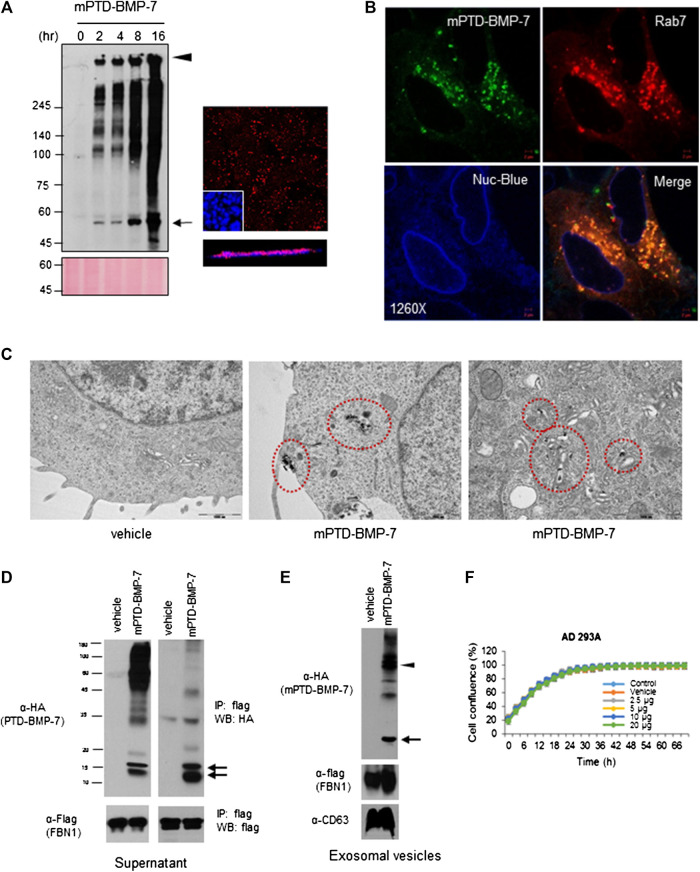
Endosomal transduction of micellized PTD-BMP-7 (mPTD-BMP-7) and exosomal secretion of bone morphogenetic protein-7 (BMP-7). **(A)** HEK 293 cells were treated with 200 ng of mPTD-BMP-7 for the indicated period, and the insoluble fraction of whole cell lysates were subjected to immunoblot analysis with anti-HA antibody **(left)**. Arrow and arrowhead denote monomeric and aggregated PTD-BMP-7, respectively. The 293 cells were treated with 200 ng of mPTD-BMP-7, and intracellular transduction was monitored by confocal microscopy **(right)**. Z-stack in blow. Scale bar, 5 μm. **(B)** The 293A cells were transiently transfected with mCherry-tagged Rab7 and treated with 200 ng of HA-tagged mPTD-BMP-7 for 3 h. Intracellular localization was observed with confocal microscopy and Nuc-blue served as nuclear staining of live cell imaging. **(C)** The 293 cells were treated with 200 ng mPTD-BMP-7, and the cells were subjected to transmission electron microscopy. Note a membrane-coated mPTD-BMP-7 in endosomes **(middle)** and Golgi **(right)**. **(D)** The 293 cells were transfected with flag-tagged N-terminal of fibrillin-1 (FBN-1) and treated with 500 ng HA-tagged mPTD-BMP-7 for 16 h. The culture medium was harvested and subjected for immunoblot analysis **(left)** and immunoprecipitation with anti-flag (FBN-1). Arrows indicate active BMP-7. **(E)** Exosomal secretion of active BMP-7 (arrow) and mPTD-BMP-7 (arrow head) with FNB-1. CD63 serves as exosomal marker. **(F)** Cellular toxicity of mPTD-BMP-7. The 293A cells were incubated in various concentrations of mPTD-BMP-7, and cell growth was monitored by JuLi real-time live cell analyzer (NanoEntek).

To examine intracellular transduction of mPTD-BMP-7, we treated mPTD-BMP-7 into 293A cells and examined the transduction by immunofluorescence and Western blot analysis in Triton-insoluble fraction because denatured PTD-BMP-7 forms insoluble aggregate in the micelle. As shown in Figure 2A, mPTD-BMP7 was successfully transduced into the cells in a time-dependent manner. Notably, mPTD-BMP-7 was continuously transduced into the cells for up to 16 h. Confocal microscopy with Z-stack sections also revealed that mPTD-BMP-7 was successfully transduced into the cytosolic space. Because the PTD-mediated protein delivery was mediated by endosomal pathway (Vendeville et al., 2004; Wadia et al., 2004), we labeled the PTD-BMP-7 with fluorescent dye and examined the intracellular localization with endosomal Rab7 (Ras superfamily of small G protein) GTPase. Indeed, transduced mPTD-BMP-7 was tightly co-localized with Rab7 in the cytosolic space (Figure 2B). To further examine the membrane-coated transduction, we labeled the PTD-BMP-7 with uranyl acetate and examined it with a transmission electron microscope. Interestingly, PTD-BMP-7 was internalized within membrane-coated intracellular vesicles and successfully transported into the Golgi apparatus (Figure 2C). These results indicate that mPTD-BMP-7 was actively transduced with endosomal pathway and transported into the trans-Golgi network.

Next, we examined whether transduced mPTD-BMP-7 is secreted to extracellular space. Because prodomain in latent BMP-7 tightly binds to FBN-1 for extracellular secretion (Gregory et al., 2005; Wohl et al., 2016), we cloned flag-tagged N-terminal of FBN1 expression vector and expressed it in 293A cells. After incubation of mPTD-BMP-7 tagged with HA in the C-terminus of BMP-7 into FBN1 expressing cells, we harvested the culture supernatant and performed immunoblot analysis. Indeed, ∼15 kd-sized active BMP-7 bands were detected compared to control (Figure 2D). When we immunoprecipitated the supernatant with FBN1, immunoblot analysis revealed that secreted BMP-7 had interacted with FBN1. Given the endosomal transduction of mPTD-BMP-7, we next collected extracellular vesicles (exosomes) and examined the secreted BMP-7. Interestingly, active BMP-7 and FBN1 were clearly detected in secreted exosomes (Figure 2E). Notably, unprocessed PTD-BMP-7 was detected in extracellular vesicles, indicating an endosomal–exosomal recycling of mPTD-BMP-7. Examining the cytotoxicity of mPTD-BMP-7, we found that mPTD-BMP-7 is not cytotoxic under excess dose exposure *in vitro* (Figure 2F). These results indicate that transduced mPTD-BMP-7 via endosomal pathway was successfully processed into mature BMP-7 and secreted with FBN1.

### Micellized Protein Transduction Domain-Bone Morphogenetic Protein-7 Counteracts Transforming Growth Factor-Beta 1–Induced Epithelial–Mesenchymal Transition Via Activation of SMAD1/5/8

**FIGURE 3:**
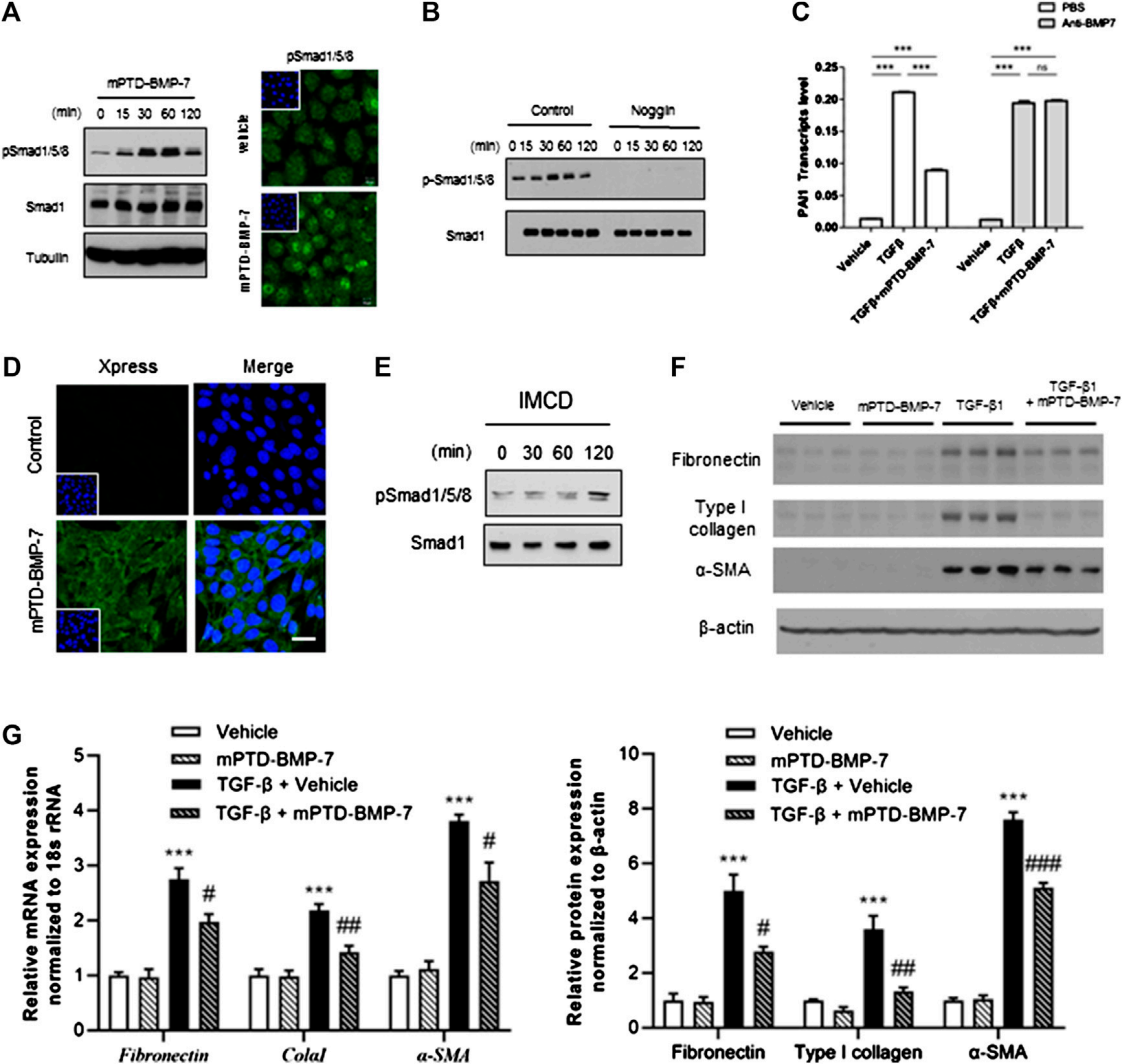
Micellized PTD-BMP-7 (mPTD-BMP-7) antagonizes transforming growth factor-beta (TGF-β)–mediated epithelial–mesenchymal transition (EMT) via activation of SMAD1/5/8. **(A)** 293A cells were treated with 100 ng of mPTD-BMP-7 and activation of SMAD1/5/8 were detected by immunoblot analysis **(left)** and immunofluorescence **(right)**. **(B)** The 293 cells transfected with control or Noggin expression vector were treated with mPTD-BMP-7, and SMAD1/5/8 phosphorylation was detected by immunoblot analysis. **(C)** mPTD-BMP-7 inhibits PAI-1 transactivation induced by TGF-β. A549 cells were incubated TGF-β (5 ng) with or without mPTD-BMP-7 for 16 h, and PAI-1 transcript abundance was detected by qRT-PCR (white). Neutralizing antibody against soluble BMP-7 abolished the effect of mPTD-BMP-7 (gray). qPCR was performed from triplicate experiment. **(D)** Intracellular transduction of mPTD-BMP-7 was detected by immunofluorescence in inner medullary collecting duct (IMCD) cells. Scale bar, 20 μm. **(E)** IMCD cells were treated with 100 ng of mPTD-BMP-7 and activation of SMAD1/5/8 were detected by Western blot analysis. (**F,G)** IMCD cells were treated with vehicle or mPTD-BMP-7 (200 ng) or TGF-β (5 ng) or combination for 3 days, and cell lysates were subjected for immunoblot analysis of fibronectin, type I collagen, and α-smooth muscle actin **(E)**. Quantitation of transcript (upper) and protein (lower) abundance of mesenchymal markers in IMCD cells, ***p < 0.001 vs. Vehicle; ^#^ *p* < 0.05 vs. TGF-β; ^##^ *p* < 0.01 vs. TGF-β; ^###^ *p* < 0.001 vs. TGF-β **(F)**.

BMP-7 immediately elicits phosphorylation of SMAD1/5/8 to counteract TGF-β signal (Wadia et al., 2004). To test whether mPTD-BMP-7 affects SMAD1/5/8 activation, we treated 100 ng of mPTD-BMP-7 and examined the phosphorylation status of SMAD1/5/8 in A549 cells. Immunoblot and immunofluorescence study revealed that mPTD-BMP-7 increased phosphorylation and its nuclear translocation of SMAD1/5/8 (Figure 3A). Because Noggin specifically binds and antagonizes active BMP-7 (Groppe et al., 2002), we next overexpressed Noggin and treated mPTD-BMP-7 to examine SMAD1/5/8 activation (Figure 3B). Indeed, mPTD-BMP-7 did not activate SMAD1/5/8 in the presence of Noggin. In TGF-ß–responsive A549 cells, the plasminogen activator inhibitor-1 (PAI-1, SERPINE1) is the typical target gene of TGF-β. When we examined the effect of mPTD-BMP-7 on TGF-β–mediated PAI-1 activation, TGF-β–activated PAI-1 transcription and the treatment of mPTD-BMP-7 suppressed PAI-1 transcript abundance induced by TGF-β (Figure 3C). Importantly, this inhibitory action counteracting TGF-β was completely abolished by the treatment of neutralizing BMP-7 antibody, indicating that mPTD-BMP-7 was successfully processed into active BMP-7 resulting in inhibition of TGF-ß.

To examine the effects of mPTD-BMP7 on EMT related to renal fibrosis, mouse inner medullary collecting duct (IMCD) cells were incubated with mPTD-BMP-7 and examined for intracellular transduction. As with the 293A cells, mPTD-BMP-7 was successfully transduced into the IMCD cells (Figure 3D) with activation of SMAD1/5/8 (Figure 3E). To determine the effect of mPTD-BMP-7 in TGF-β–mediated EMT, we treated TGF-β in combination with mPTD-BMP-7 and examined EMT markers in IMCD cells. The TGF-β treatment increased expression of typical mesenchymal genes including fibronectin, type I collagen, and α-smooth muscle actin, while mPTD-BMP-7 largely blocked TGF-β–mediated EMT (Figure 3F). Those protein expressions of mesenchymal markers were closely correlated to the transcript abundance (Figure 3G). These results indicate that mPTD-BMP-7 inhibits the TGF-β–mediated EMT process by the activation of SMAD1/5/8.

### Effect of Micellized Protein Transduction Domain-Bone Morphogenetic Protein-7 on the Unilateral Ureteral Obstruction Renal Fibrosis Model

**FIGURE 4:**
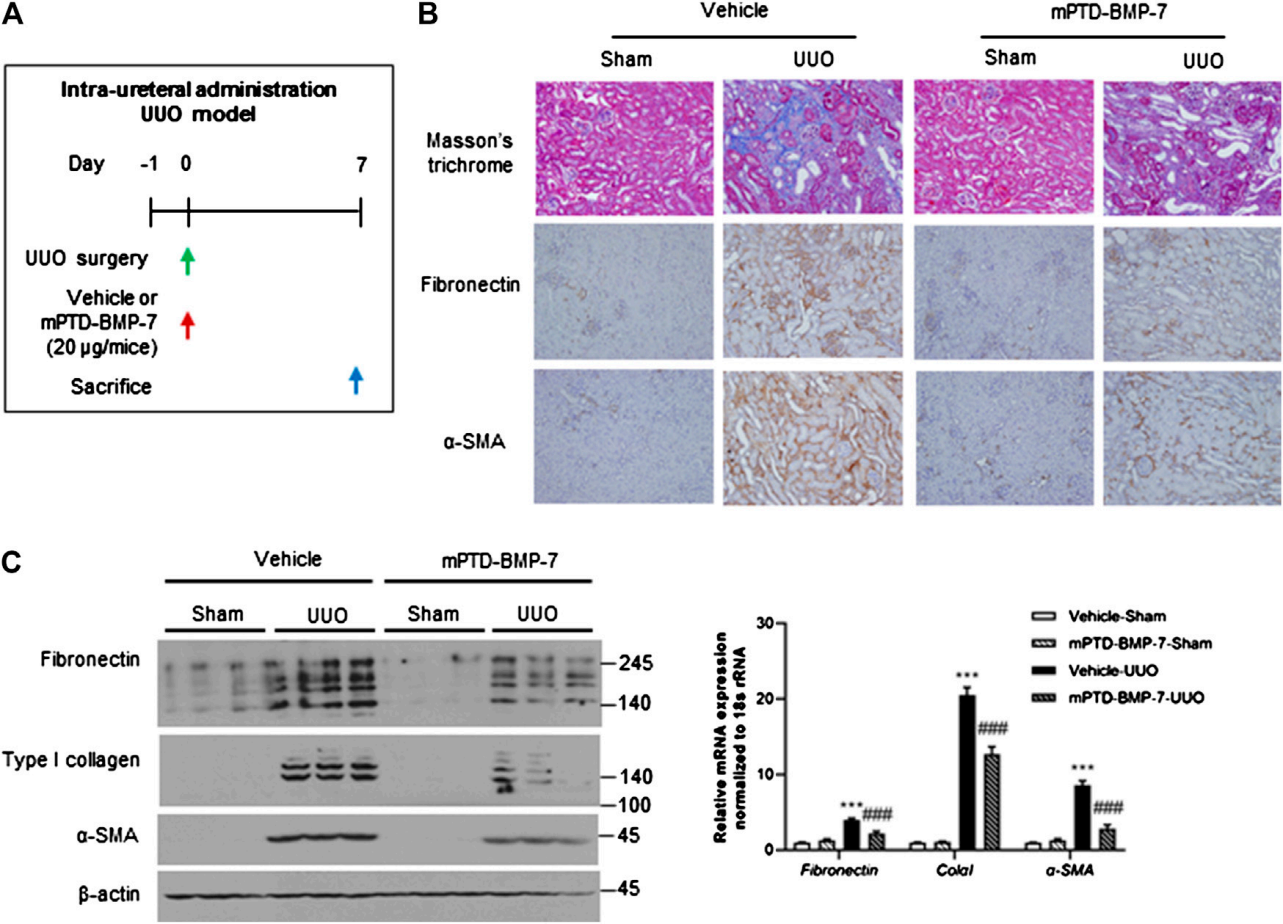
Micellized PTD-BMP-7 (mPTD-BMP-7) attenuates renal fibrosis in mouse unilateral ureter obstruction (UUO) model. **(A)** Schematic diagram for mouse UUO experiment with intra-ureteral injection of mPTD-BMP-7. **(B)** Masson’s trichrome stain and immunohistochemical staining of fibronectin and α-smooth muscle actin in UUO kidneys. **(C)** Immunoblot analysis for mesenchymal markers in mouse UUO kidney **(left)** and quantitative data **(right)**. ***p < 0.001 vs. Vehicle-Sham; ^###^ *p* < 0.001 vs. Vehicle-UUO.

UUO is a well-established model for renal fibrosis, given the pivotal role of TGF-β signaling during UUO-induced EMT (Miyajima et al., 2000; Ma et al., 2003). To determine the anti-fibrotic effect of mPTD-BMP-7 *in vivo*, we designed a mouse UUO model using intraurethral injection of mPTD-BMP-7 (Figure 4A). Masson’s trichrome and immunohistochemistry revealed that the UUO induced severe tubule-interstitial fibrosis, while the intraurethral injection of mPTD-BMP-7 reduced UUO-induced renal fibrosis (Figure 4B). Immunohistochemistry showed that fibronectin and α-smooth muscle actin specifically deposited in the interstitial space of kidney by UUO and mPTD-BMP-7 largely decreased interstitial fibronectin and α-smooth muscle actin. To quantitate mesenchymal markers, we examined protein and transcript abundances in mouse kidney samples. Consistently, both mRNA and protein abundances of mesenchymal genes were significantly increased by UUO, and the EMT progression was relieved by mPTD-BMP-7 injection (Figure 4C). Multiple isoforms due to the alternative splicing of fibronectin and type I collagen were increased by UUO, and all isoforms were suppressed by the treatment of mPTD-BMP-7. Although intraurethral injection was effective in mouse model, pharmacologic approach via urethra is very limited in human. To determine the route of administration for clinical application, we next examined therapeutic effects of mPTD-BMP-7 by repeated intravenous injection in the mouse UUO model. However, we found the therapeutic potential of mPTD-BMP-7 disappointing with intravenous administration in the UUO model, probably due to limited delivery into the kidney (Supplementary Figure S1). These results suggest that a different route of administration of mPTD-BMP-7 is required for kidney.

### Application of Micellized Protein Transduction Domain-Bone Morphogenetic Protein-7 by Angiography on Unilateral Ureter Obstruction–Induced Renal Fibrosis Model

**FIGURE 5:**
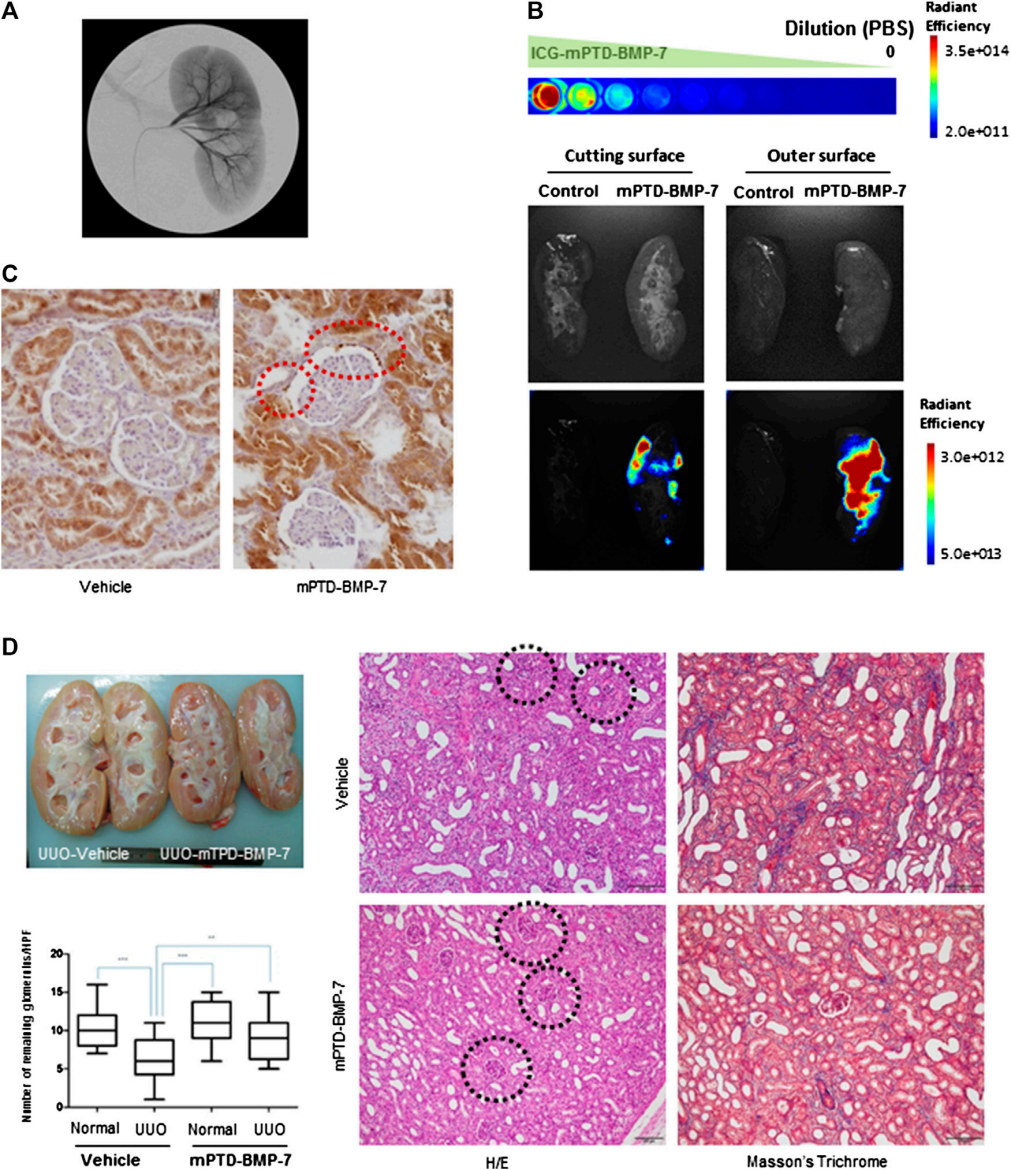
Interventional administration of micellized PTD-BMP-7 (mPTD-BMP-7) via renal artery attenuates renal fibrosis in pig unilateral ureter obstruction. **(A)** Renal angiography after insertion of micro-catheter into renal artery. **(B)** ICG-labeled mPTD-BMP-7 was serially diluted with PBS for fluorescence quantitation **(upper)**. Gross **(middle)** and fluorescence **(lower)** images of pig kidney administrated vehicle or mPTD-BMP-7. The pig kidneys were divided into half by longitudinal sections for inner medullary **(left)** and outer cortical **(right)** areas. **(C)** Immunohistochemical staining of pig kidney using polyclonal antibody against prodomain of bone morphogenetic protein-7. Note the increased expression level of bone morphogenetic protein-7 in proximal tubules and multiple granule-like foci in the Bowman’s space in mPTD-BMP7 administrated kidney (red circles). Scale bar, 20 μm. **(D)** Gross **(upper left)** and histologic **(right)** images of vehicle or mPTD-BMP-7 administrated pig kidney. Black circles denote remaining glomerulus. The number of remaining glomeruli was counted in randomly selected 20 high power fields **(lower left)**.

Recently, vascular intervention under image guidance has rapidly expanded to treat specific organs. We attempted to deliver mPTD-BMP-7 by catheter-directed intra-arterial administration into pig kidney through renal artery. We found that this provides a novel route of administration to deliver mPTD-BMP-7 into the pig kidney (Figure 5A). To determine the delivery potential of renal arterial intervention, we labeled PTD-BMP-7 with ICG and formulated in micelle. To administrate for a sufficient period, the ICG-labeled mPTD-BMP-7 (100 μg) was diluted in saline (100 ml) and injected into the renal artery with a 2.1 F microcatheter for 2 h period. After 3 h intra-arterial administration of mPTD-BMP-7, the pig was sacrificed and the kidney was examined with an *in vivo* bioluminescent imaging system to determine whether mPTD-BMP-7 was delivered into pig kidney. Indeed, ICG-labeled mPTD-BMP-7 was successfully delivered into the kidney parenchyma (Figure 5B). Notably, the fluorescence intensity of the outer surface was stronger than the inner medullar space, suggesting that mPTD-BMP-7 is mainly delivered into the renal cortex, consisting of glomeruli and tubules. Considering a depth limitation of fluorescence image, more than 10 μg of mPTD-BMP-7 was delivered into the pig kidney. To further determine renal distribution of mPTD-BMP-7, we performed immunohistochemical staining with two different antibodies to detect epitopes for prodomain and active domains of BMP-7, respectively. Consistent with a critical function of BMP-7 in kidney (Godin et al., 1998; Gould et al., 2002; Tomita et al., 2013), endogenous BMP-7 is highly expressed in kidney parenchyma, especially in proximal tubules (Figure 5C; Supplementary Figure S2A). As both antibodies detected BMP-7 similarly, expression levels of BMP-7 in proximal tubules were increased in mPTD-BMP-7 treated kidney compared to vehicle. In particular, numerous BMP-7–positive granule-like foci were clearly noted following the administration of mPTD-BMP-7 in Bowman’s space and the parietal layer of the glomerular capsule. Renal transduction of mPTD-BMP-7 was also verified with Western blot analysis from the pig kidney (Supplementary Figure S2B). These results indicate that the interventional approach via renal artery successfully delivered mPTD-BMP-7 to the kidney parenchyma and that mPTD-BMP-7 is actively transported from the glomerular capillaries to Bowman’s space and proximal tubules, probably by endosomal uptake and exosomal transport.

To examine whether the interventional approach is effective in the UUO model, we next made a laparoscopic porcine UUO model and administrated mPTD-BMP-7 to the kidney for 2 h with the use of infusion set via the renal artery. After 1 week of UUO surgery, the pig kidneys were examined grossly and histologically (Figure 5E). Grossly in hemi-section, the mPTD-BMP-7–treated kidney was less swollen and more pinkish in color. Histologically, UUO induced severe interstitial fibrosis with tubular atrophy, while the mPTD-BMP-7 treatment decreased fibrosis and tubular atrophy. Interestingly, the number of existing glomerulus in the UUO kidney was increased by mPTD-BMP-7 administration compared to control. To revalidate the porcine model with intervention, we have performed repeated experiments and obtained similar results (Supplementary Figure S2C).

## Discussion

Although the pivotal role of TGF-β–induced EMT in various fibrotic diseases is well known, an effective therapeutic is still lacking. Small molecule inhibitors and antibodies targeting TGF-β receptors are under active development. For example, Galunisertib targets kinase activity of TGF-β receptor I for treating cancer (Huynh et al., 2019). Several antibodies targeting ligand-receptor interaction have also been developed. However, clinical application of those therapeutics for chronic diseases is very limited due to on-target toxicities, such as cardiac toxicity (de Gramont et al., 2017).

As a well-known endogenous TGF-β inhibitor, BMP-7 comprises an alternative approach against TGF-β–induced fibrotic diseases including kidney (Zeisberg et al., 2003b; Manson et al., 2015). While the TGF-β specifically induces and binds to the extradomain-A isoform of fibronectin resulting in myofibroblastic differentiation and α-smooth muscle actin expression in UUO model (Serini et al., 1998; Yokoi et al., 2004), mPTD-BMP-7 decreased all isoforms of fibronectin and type I collagen. Further study is required to determine BMP-7’s anti-fibrotic effect on extradomain-A fibronectin and thus delineate the mechanistic link between TGF-β and BMP-7. In addition to its effectiveness, BMP-7 therapeutics for fibrosis provide long-term safety with excess dose, a significant clinical advantage (Zeisberg et al., 2003a). In addition, transgenic overexpression of systemic BMP-7, including kidney and liver, was tolerable in mice with normal renal function up to 1 year (Wang et al., 2006).

Similar to TGF-β, BMP-7 is generated from a precursor polypeptide that is proteolytically cleaved, generating a homo-dimeric N-terminal prodomain and disulfide-linked mature dimer (Gregory et al., 2005; Robertson and Rifkin, 2016). The prodomains carrying mature BMP-7 tightly interact with FBN1, forming a latent BMP-7 which is secreted into extracellular space and providing long-range signaling activities *in vivo* (Gregory et al., 2005; Wohl et al., 2016). Although latent BMP-7 is largely regarded as biologically inactive, the prodomain of the TGF-β superfamily is critically required for the long-range action due to its serving as a niche for growth factors, especially in clinical and *in vivo* (Sengle et al., 2011; Sengle and Sakai, 2015). Interestingly, active TGF-β and BMP-7 in the latent complex directly interact with TGFBRII and BMPRII, respectively (Sengle et al., 2008; Campbell et al., 2020). Furthermore, those latent TGF-β and BMP-7 are biologically active without being released from the prodomain by extracellular proteases (Sengle et al., 2008; Campbell et al., 2020). While most *in vitro* and *in vivo* studies have been conducted with rhBMP-7 without prodomain, the bioavailability and physiological relevance of latent BMP-7 is not well understood.

In this study, we designed a recombinant BMP-7 including prodomain and formulated in micelle (mPTD-BMP-7) to increase intracellular transduction and long-range signaling activity *in vivo*. We found that mPTD-BMP-7 successfully activates SMAD1/5/8 and inhibits TGF-β–induced PAI-1 transactivation. Noggin expression or neutralizing antibody abolished those effects, indicating that active BMP-7 is being secreted following the transduction of mPTD-BMP-7. Importantly, mPTD-BMP-7 is transduced into the cells by endosomal transport and secreted with FBN1 in exosomes. The fluorescent-labeled exosomes injected into circulating blood could secrete into the urine (Cheng et al., 2012). Therefore, administration of mPTD-BMP-7 through renal artery can be delivered into the Bowman’s space and collecting tubules by exosomal transport.

In conclusion, we report that prodrug BMP-7 provides an alternative therapeutic against TGF-β–mediated fibrotic disease with minimal toxicity. Furthermore, interventional delivery of mPTD-BMP-7 via renal artery increased bioavailability into the kidney. Further pharmacologic and preclinical studies are required for clinical therapeutics for renal fibrosis.

## Materials and Methods

### Cell Lines and Expression Vectors

HEK293, A549 cells (obtained from ATCC) and 293A cells (Thermo Fisher) were routinely cultured in DMEM medium containing 10% FBS. IMCD cells (a gift from professor JP Lee at Boramae Hospital) were cultured in a DMEM/F12 medium with 10% FBS. Human Noggin and FBN-1 (a.a. 1–1,647) expression vectors tagged with flag or 6× His were generated from cDNA of 293 cells. Fluorescence mCherry-tagged Rab7 expression vector was kindly provided by S. Y. Kim (KNCC, Korea). The transfection was performed by Lipofectamine 2000 according to the manufacturer’s protocol (Invitrogen).

### Micellized Protein Transduction Domain-Bone Morphogenetic Protein-7 Preparation

The bacterial expression vectors for HA-tagged PTD-BMP-7 and protein purification were described previously (Vendeville et al., 2004; Wadia et al., 2004; Kim et al., 2014). The denatured polypeptide was micellized with filtered 0.1% egg lecithin (BOC Sciences) using bath sonication. For *in vivo* fluorescent imaging, PTD-BMP-7 was labeled with ICG (ICG-sulfo-OSu, Dojindo Molecular Technologies). Alternatively, PTD-BMP-7 was fluorescent labeled with the Alexa-Fluor-488 protein labeling kit (Invitrogen) for immunofluorescence study. For cellular transduction, mPTD-BMP-7 was directly treated into the culture medium. Particle size of mPTD-BMP-7 was determined by direct light scattering (ELSZ-200ZS, Otsuka Electronics).

### Western Blot Analysis and Immunofluorescence

For the Western blot analyses, protein extracts were prepared in Triton ×-100 lysis buffer. Antibodies against HA (901501, BioLegend, 1:5,000), BMP-7 (ab84684, Abcam, 1:2,000), SMAD1 (#9743S, Cell Signaling, 1:2,000), phospho-SMAD1/5/8 (#13820, Cell Signaling, 1:2,000), CD63 (sc-5275, Santa Cruz, 1:1,000), fibronectin (a0245, Dako, 1:5,000), type I collagen (1310-01, SouthernBiotech, 1:500), alpha-smooth muscle actin (ab5694, Abcam, 1:1,000), ß-actin (a5441, Sigma-Aldrich, 1:5,000), and tubulin (LF-PA0146, AbFrontier, rabbit polyclonal, 1:5,000) were obtained from commercial vendors. Protein abundance in immunoblot analysis was quantitated by the publicly available ImageJ program. For immunofluorescent analysis, the cells were treated with mPTD-BMP for 16 h and intracellular transduction of recombinant protein was detected against anti-HA monoclonal antibody and anti-mouse IgG-conjugated Alexa-Fluor-488 secondary antibody, as described previously (Kim et al., 2014). The 293A cells were transfected with mCherry-Rab7 for a 48 h period, and Alexa-488 tagged mPTD-BMP-7 was treated to cells for 3 h followed by nuclear stain with NucBlue Probe (Thermo Fisher, R37605) for live cell imaging. Cellular fluorescence was monitored using confocal microscopy (Zeiss LSM780). Rabbit polyclonal antibody detecting prodomain of BMP-7 was generated by synthetic peptide (GLIGRHGPQNKQP) followed by affinity purification (Labfrontier). Functional blocking of secreted BMP-7 was performed by directly adding 10 *µ*g/ml neutralizing antibody against BMP-7 (MAB3542, R&D Systems) to normal culture medium.

### quantitative Polymerase Chain Reaction and Primers

Total RNA was isolated using TRIzol reagent (Invitrogen) following the manufacturer’s protocol. The SuperScript III synthesis kit (Invitrogen) was used to generate cDNA. Real-time quantitative PCR analysis was performed with an ABI-7300 instrument under standard conditions and SBGR mix (*n* = 3). The ΔCt value expression from each sample was calculated by normalizing with GAPDH or 18S rRNA. The relative RNA amount of PAI1 were normalized to GAPDH, and the relative RNA amount of Fibronectin, ColaI, and aSMA were normalized to 18s rRNA. Primer specificity and PCR process were verified by dissociation curve after PCR reaction. The primer sequences for qPCR are described separately in Supplementary Data.

### Transmission Electron Microscopy and Scanning Electron Microscopy

The prepared mPTD-BMP-7 was directly subjected to field emission scanning electron microscope (Merlin, Zeiss). For transmission electron microscopy (TEM), PDT-BMP-7 was stained with 5% uranyl acetate for 10 min, and prepared for micelle. 293A cells were treated with uranyl acetate–stained mPTD-BMP-7 for various time periods, and the cells were fixed with 2.5% glutaraldehyde. Cellular transduction of mPTD-BMP-7 was monitored by TEM (JEM-1011, JEOL, Peabody, MA, United States).

### Histological Examination and Immunohistochemistry

For histological and immunohistological examination, serial paraffin sections were stained with H&E for routine morphological observation. For immunohistochemical staining, tissue sections in microslides were deparaffinized with xylene, hydrated in serial dilution of alcohol, and immersed in 3% hydrogen peroxide. The tissue sections were incubated with protein blocking agent for 20 min at room temperature and then incubated overnight at 4°C with primary antibody. After washing with PBS three times, the sections were incubated with a biotinylated secondary antibody (Immunotech) and streptavidin conjugated to horseradish peroxidase for 20 min at room temperature. The chromogen was developed for 5 min with 3-amino-9-ethylcarbazole (AEC) followed by counterstain with Meyer’s hematoxylin.

### Exosomal Purification

The 293 cells transiently transfected with flag-tagged FBN-1 expression vectors were treated with 500 ng of mPTD-BMP-7 in FBS-free DMEM (0.1% BSA) for a 16-h period. The culture medium was centrifuged at 800 rpm for 3 min to remove cell debris followed by 0.2 μm filtration. Two volumes of culture medium were mixed with Exo-spin buffer (EX01, Cell Guidance Systems). After 16 h, the mixture was centrifuged at 13,000 rpm for 60 min, and exosomal pellets were obtained.

### Animal Experiments

All animal experiments were performed in accordance with the Institutional Animal Care and Use Committee of the Yonsei University and approved by the Animal Care Committee of the Yonsei University College of Dentistry and National Cancer Center Research Institute. Six-week old C57BL/6 mice were anesthetized with intraperitoneal injection of tiletamine/zolazepam (Zoletil^®^)-xylazine (Rompun^®^) mixture (1 mg/kg) placed on a heating surgical pad. The mice underwent either a sham operation or unilateral ureteral ligation with 4-0 suture silk through an abdominal midline incision. A single dose of vehicle (40 μl) or 20 μg mPTD-BMP-7 was injected into the urether above the UUO ligation. Separately, UUO mice were administrated 20 μg of mPTD-BMP-7 or vehicle via tail vein every day for 1 week. The UUO mice were euthanized at day 7, and both obstructed or unobstructed counterlateral kidney were harvested for paraffin-embedded sections. Renal fibrosis was determined by interstitial collagen deposition, immunostaining for α-smooth muscle actin, and fibronectin.

For intra-arterial administration of mPTD-BMP-7, a female 12-week-age Yorkshire pig was used. The right femoral artery access was used in all cases for pig. The left renal artery was selected with a 5.0-F Kumpe catheter (Cook Inc.), and renal angiography was obtained. Under the roadmap image, a coaxial 2.4 F microcatheter (Progreat, Thrumo Medical) was gently advanced into the main renal artery. Immediately after the catheter insertion, total 100 ml of 0.9% saline mixed with ICG-labeled mPTD-BMP-7 100 μg was infused into the left renal artery over 2 h (50 ml/h) with infusion set (Sungshim Medical). After 3 h, the pig was sacrificed to harvest both kidneys for fluorescent imaging. Transduction of mPTD-BMP-7 into the pig kidney was harvested for *ex vivo* fluorescence imaging analysis and immunohistologic analysis. Fluorescence images were acquired by VISQUE^®^ InVivo Smart (Vieworks, Anyang, South Korea) and were analyzed by CleVue^™^ software (Vieworks, Anyang, South Korea). Total signal intensity (*cm^2^) was normalized by subtraction of control (counter lateral) signal intensity. After the fluorescence imaging analysis, kidney specimens were fixed in 10% formalin for paraffin sections. To examine the intra-arterial injection of mPTD-BMP-7 in pig UUO, unilateral urethral obstruction (left side only) was done on the pig using laparoscopic approach under general anesthesia. The next day, 100 μg of mPTD-BMP-7 was administrated via renal artery for 2 h. After 7 days from mPTD-BMP-7 treatment, the pig was euthanized, and both kidneys were harvested for histologic analysis with H&E stain and Masson’s Trichrome stain.

## Data Availability Statement

The datasets presented in this article are not readily available because the data that support the findings of this study are available from the corresponding authors upon reasonable request. Requests to access the datasets should be directed to Nam Hee Kim, MIGO77@yuhs.ac.

## Ethics Statement

The animal study was reviewed and approved by the Institutional Animal Care and Use Committee of the Yonsei University.

## Author Contributions

SK and C-HJ performed experiment; S-HS, JEU, JSY, DH, ESC, and BYN supported *in vitro* and *in vivo* experiments. MK and JY performed *in vivo* image analysis. HSK, JIY, M-DK, NHK, and T-HY planned all experiments, analyzed the data, and wrote the manuscript.

## Funding

This work was supported by grants from the National Research Foundation of Korea (NRF-2014R1A2A1A11050098, 2016R1E1A1A01942724, NRF-2017R1A2B3002241, NRF-2017R1A2B4005720, NRF-2019R1A2C2084535) funded by the Korea government (MSIP), a grant from the National Research Foundation of Korea (NRF-2020R1I1A1A0107297711) funded by the Korea government (MOE). M-DK was supported by a grant from WITHHEALTHCARE (Grant No. 2018-31-1196).

## Conflict of Interest

JIY is an inventor of the patent related to this work filed by MET Life Sciences Co., Ltd. (Korean Patent Application Number: 10-2019-0076443, PCT Application Number: PCT/KR2020/007011). NHK, HSK, T-HY, and JIY are the founders of MET Life Sciences Co., Ltd. and shareholder.

All other authors declare that they have no competing interests.
