# Supplementary material for: Micellized Protein Transduction Domain-Bone Morphogenetic Protein-7 Efficiently Blocks Renal Fibrosis Via Inhibition of Transforming Growth Factor-Beta–Mediated Epithelial–Mesenchymal Transition
Source: Front Pharmacol. 2020 Nov 19;11:591275. doi: 10.3389/fphar.2020.591275 (PMC7751754; doi:10.3389/fphar.2020.591275)
Supplement: Supplementary file 1 [file Presentation1_v1.PPTX]

## Slide 1
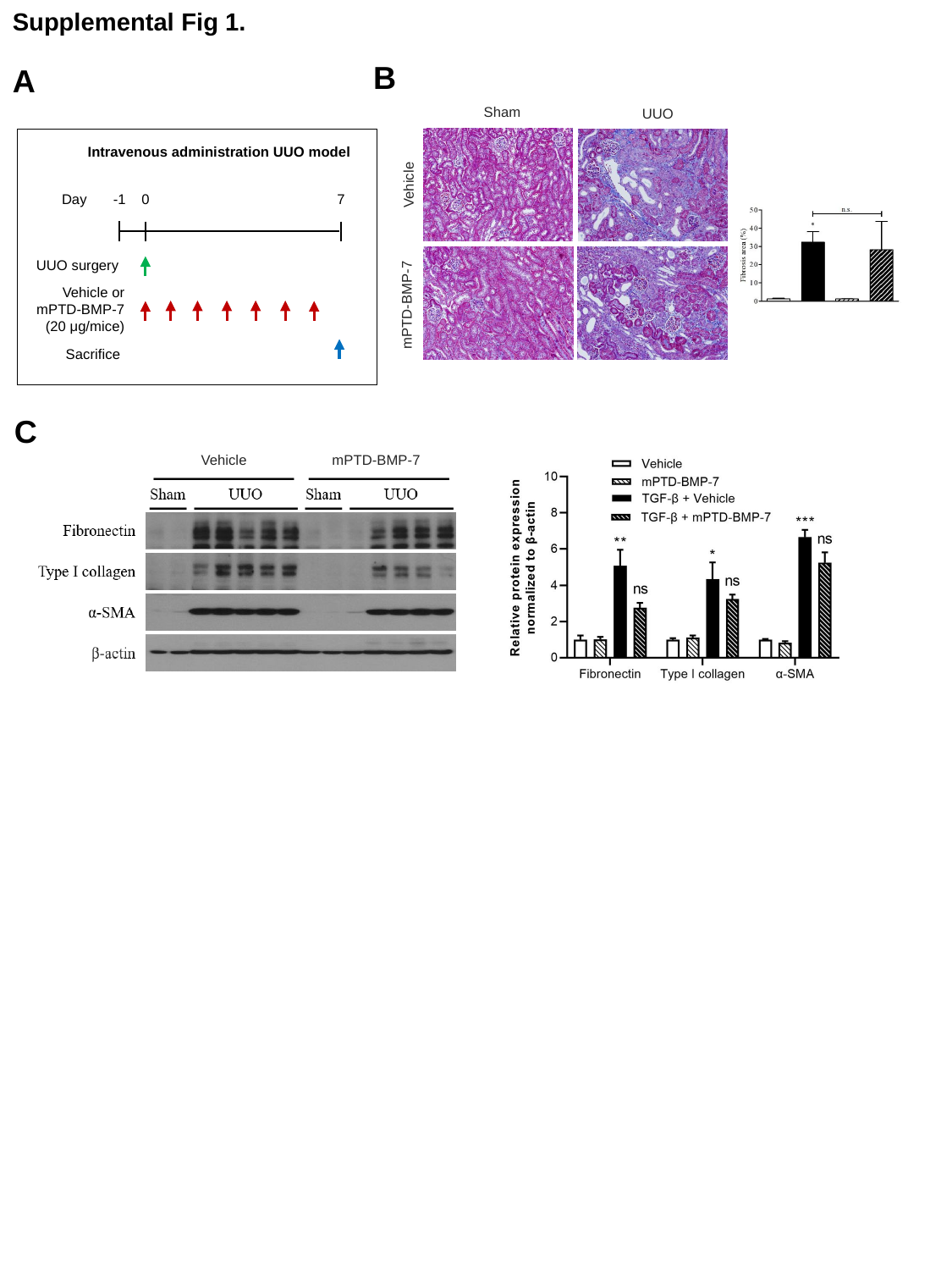

Supplemental Fig 1.
B
A
Sham
UUO
Intravenous administration UUO model
7
0
Day
-1
UUO surgery
Vehicle or mPTD-BMP-7
(20 μg/mice)
Sacrifice
Vehicle
mPTD-BMP-7
C
Vehicle
mPTD-BMP-7

## Slide 2
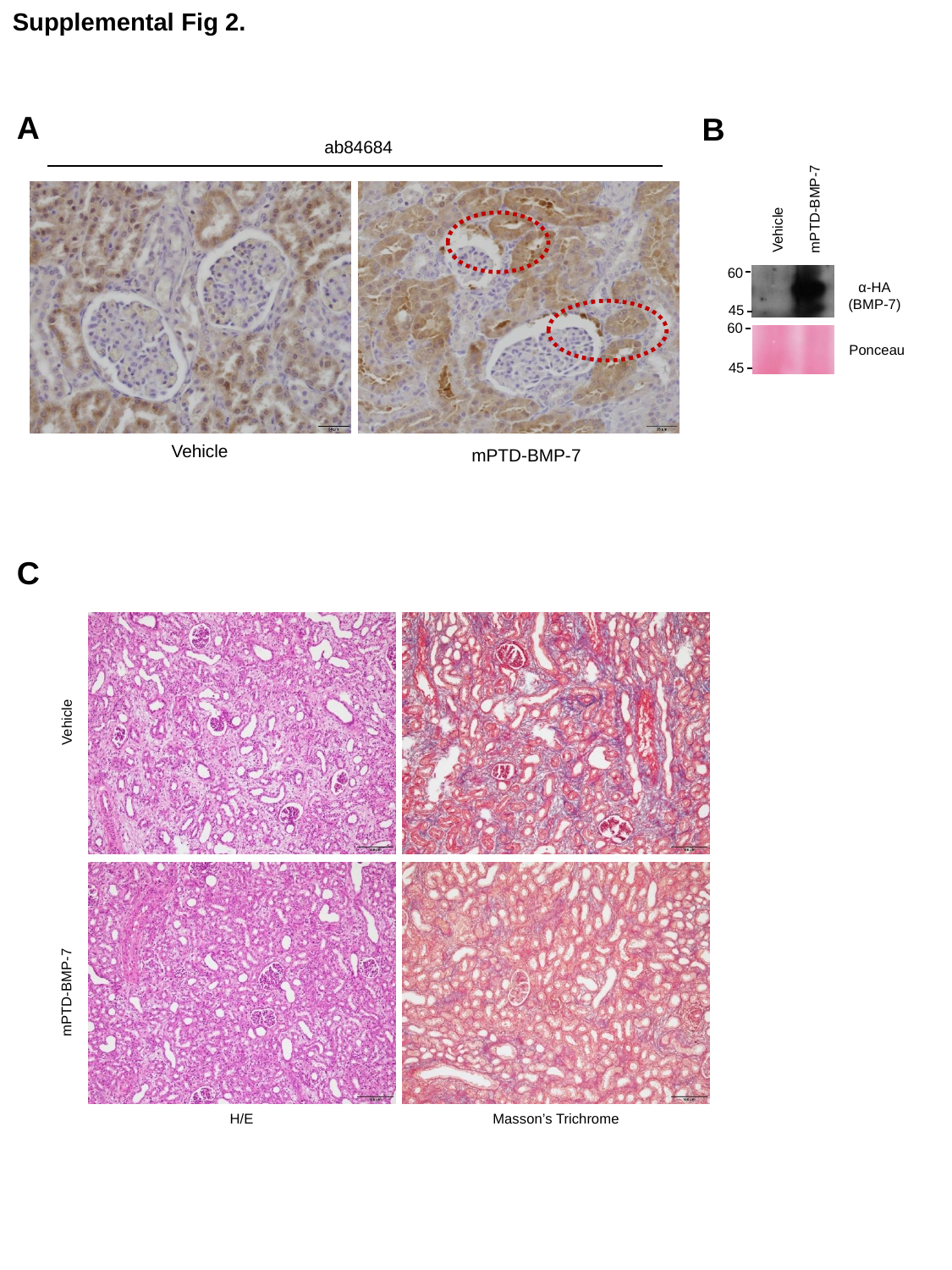

Supplemental Fig 2.
A
B
ab84684
Vehicle
mPTD-BMP-7
mPTD-BMP-7
Vehicle
60
α-HA
(BMP-7)
45
60
Ponceau
45
C
Vehicle
mPTD-BMP-7
H/E
Masson’s Trichrome

## Slide 3
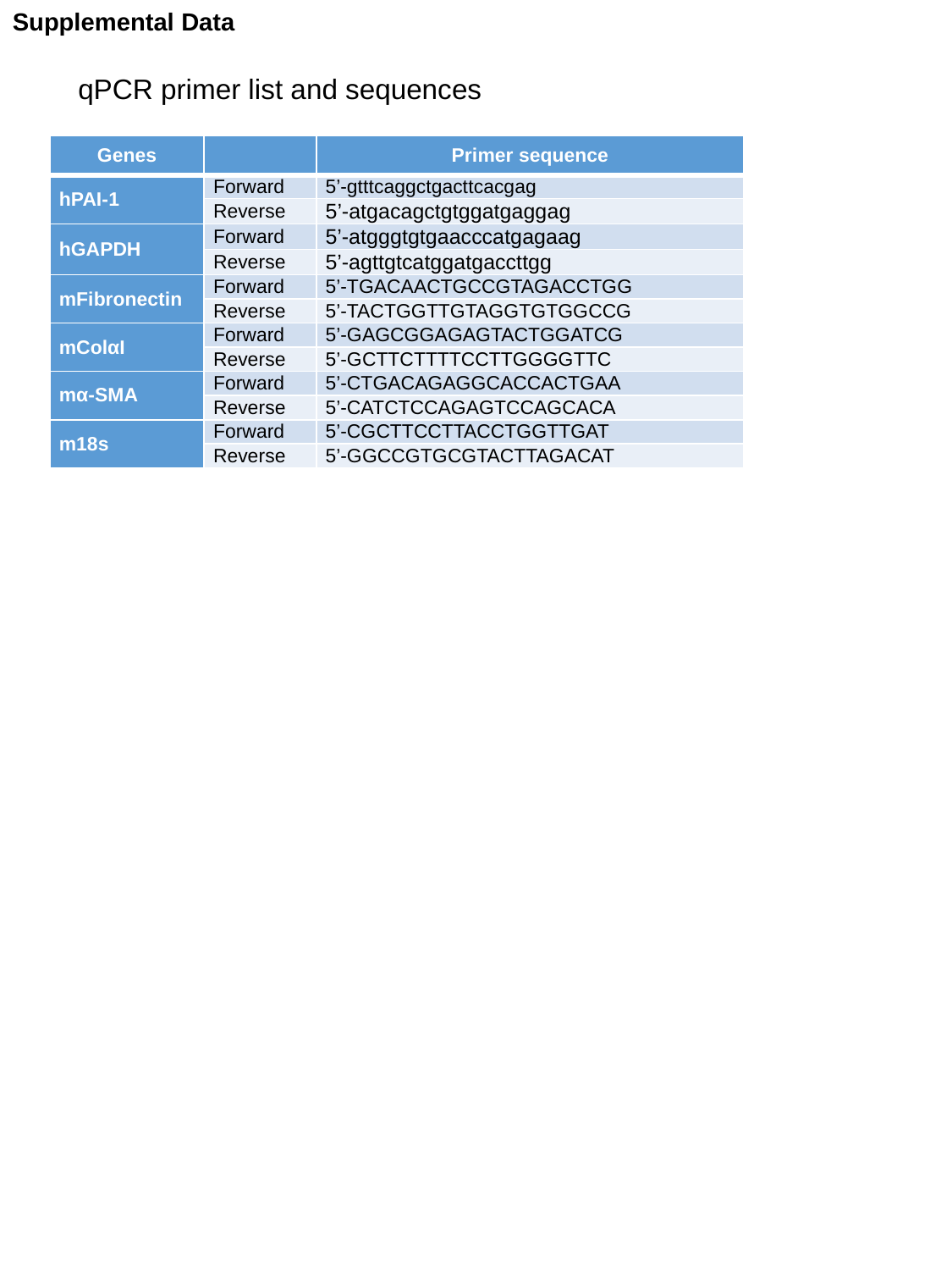

Supplemental Data
# qPCR primer list and sequences
| Genes | | Primer sequence |
| --- | --- | --- |
| hPAI-1 | Forward | 5’-gtttcaggctgacttcacgag |
| | Reverse | 5’-atgacagctgtggatgaggag |
| hGAPDH | Forward | 5’-atgggtgtgaacccatgagaag |
| | Reverse | 5’-agttgtcatggatgaccttgg |
| mFibronectin | Forward | 5’-TGACAACTGCCGTAGACCTGG |
| | Reverse | 5’-TACTGGTTGTAGGTGTGGCCG |
| mColαI | Forward | 5’-GAGCGGAGAGTACTGGATCG |
| | Reverse | 5’-GCTTCTTTTCCTTGGGGTTC |
| mα-SMA | Forward | 5’-CTGACAGAGGCACCACTGAA |
| | Reverse | 5’-CATCTCCAGAGTCCAGCACA |
| m18s | Forward | 5’-CGCTTCCTTACCTGGTTGAT |
| | Reverse | 5’-GGCCGTGCGTACTTAGACAT |
